# Adult Patients with Difficulty Swallowing Oral Dosage Forms: A Systematic Review of the Quantitative Literature

**DOI:** 10.3390/pharmacy11050167

**Published:** 2023-10-19

**Authors:** Anne Harnett, Stephen Byrne, Jennifer O’Connor, Declan Lyons, Laura J. Sahm

**Affiliations:** 1 University Hospital Limerick, V94 F858 Limerick, Ireland; anne.harnett1@hse.ie (A.H.); declan.lyons@hse.ie (D.L.); 2 Pharmaceutical Care Research Group, School of Pharmacy, University College Cork, T12 K8AF Cork, Ireland; stephen.byrne@ucc.ie (S.B.);; 3 Pharmacy Department, Mercy University Hospital, Grenville Place, T12 WE28 Cork, Ireland

**Keywords:** solid oral dosage form (SODF), medicines administration, difficulty swallowing, dysphagia, medicines manipulation, inpatient

## Abstract

The aim of this systematic review was to identify and critically appraise the available evidence regarding solid oral dosage forms (SODFs), e.g., tablets, and challenges regarding the oral administration of medicine to inpatients in a variety of healthcare settings such as (1) hospitals, (2) nursing homes and (3) long-term stay units (LTSUs). A literature search was undertaken in September 2021 and repeated in June 2023 in the following databases: PubMed, EMBASE, CINAHL, Scopus, Web of Science, The Cochrane Library, PsycINFO and ProQuest. A Microsoft Excel^®^ spreadsheet was devised to collate the following data from each eligible study: study author and year, country, number of participants, title, duration (follow-up period), study design, inclusion and exclusion criteria, method and data collection, relevant outcomes, and key findings. A total of 3023 records were identified, with 12 articles being included in the final systematic review. Seven of the twelve studies reported on the prevalence of difficulties swallowing SODFs, which varied from 10–34.2%. Nine of the twelve studies reported the methods used to manipulate SODFs, with the most reported method being tablet crushing. Given the prevalence of swallowing difficulties and the subsequent crushing of medicines in response to this, it is evident that concerns should be raised regarding the potential for a medication administration error to occur.

## 1. Introduction

One of the most prevalent and successful ways in which to prevent, manage and cure disease and ill health is using medication [1]. However, published research has shown that despite the clear advantages of medicines in helping patients, many patients do not take their medicines as prescribed, a concept termed by healthcare professionals as non-adherence [2]. Non-adherence may be unintentional or intentional [2]. Unintentional non-adherence can occur due to a lack of capacity or resources to take medications, e.g., patients forgetting to take medication. In older adults for example, unintentional adherence may be due to cognitive or psychological problems, visual or motor impairments, or a lack of adequate support structures [3]. Intentional non-adherence is associated with a decision, which may be based upon the perceived need for the medicine and may also involve the modification of the medicine or the doses taken [4,5]. The constructs underlying intentional non-adherence are varied, but can include difficulty swallowing the medicine, amongst other reasons. There are many negative consequences of non-adherence, including disease progression for the patient and increased healthcare utilisation and cost [6]. A review of interventions to improve adherence advises that any intervention should address specific medication adherence barriers for the individual patient [7].

Medicines are most commonly administered via the oral route [8]. However, patients may have trouble taking their medicines in this way due to a diagnosed or perceived swallowing difficulty [9]. Dysphagia is the medical term for swallowing difficulties, and while patients with dysphagia (PWDs) may have problems swallowing certain foods or liquids, others may not be able to swallow at all [10]. Swallowing difficulties may be overcome using routes of administration other than oral, such as the transdermal parenteral route. However, the number of medicines suitable for administration via routes other than oral is limited and the parenteral route may not be suitable for long-term administration. Another option is to use an enteral feeding tube (EFT) for the administration of medicines, e.g., a naso-gastric (NG) or gastrostomy tube. EFTs are often inserted when the risk of the aspiration of medicines or food is high when taken via the oral route. In many instances, however, the patient may not have a formal diagnosis, but rather may find that the act of swallowing presents challenges due to sensations such as a feeling of choking, gagging, or a fear that the medicine may get stuck [11]. These patients and/or their carers may seek to overcome these challenges by modifying the tablet or capsule. This is known as solid oral dosage form (SODF) modification. In many instances, the tablet will be crushed or split, or the capsule might be opened, and the contents removed for easier administration. In healthcare settings, e.g., hospitals and nursing homes, most patients have their medications administered via a healthcare professional (HCP), where again the modification of SODF takes place to facilitate administration [12,13]. In these settings, non-adherence is not as much of a problem, but SODF modification still occurs to facilitate the administration of oral medicines to patients with a swallowing difficulty or an EFT.

Whilst SODF modification presents a pragmatic and simple solution, it does not come without its own risks and challenges. These include the fact that any modification of a SODF from its authorised form may change the absorption, distribution, metabolism and or elimination (ADME) profile of that authorised product [14]. This is particularly problematic for oral sustained-release or enteric-coated formulations, leading to unintended consequences such as therapeutic failure or toxicity [15]. Patients, carers and HCPs can be exposed to cytotoxic, hormonal or teratogenic medicines if powder aerosolisation results from the crushing process [14]. Irritation to the respiratory tract, skin or mucous membranes can also occur. Additionally, crushing SODFs is almost always outside the terms of the marketing authorisation for the medicinal product [16]. Patients without an EFT face the difficulty of ingesting a manipulated SODF, which may be unpalatable and result in the use of vehicles that are unsuitable [14]. Medication errors and non-adherence to medicine regimens is a significant cost burden to health services [6]. Whilst a significant volume of research has been conducted in the paediatric population [17,18,19], the aim of this systematic review is to identify and critically appraise the available evidence regarding oral medicine administration to adult inpatients in a variety of healthcare settings, such as (1) in hospitals, (2) nursing homes and (3) long-term stay units (LTSUs).

The objectives of the review are as follows: (i) to examine the prevalence of swallowing difficulties, (ii) to describe the medication management practices used to administer oral medicines, and (iii) to examine the prevalence and type of medication administration errors associated with solid oral dosage form (SODF) manipulation.

## 2. Search Strategy

The search strategy was designed in PubMed using a combination of relevant text terms and Medical Subject Headings (MESH) terminology related to (i) hospital inpatients, (ii) oral dosage forms, (iii) methods of modification and (iv) difficulty swallowing. A comprehensive list of free text terms, devised to describe ‘methods of modification’, was used as per [14] with the addition of one free text term ‘open’, to include studies that involved the opening of capsules. Similar search strategies with MeSH terms mapped to appropriate key words were used for the search of additional databases.

A comprehensive, systematic literature search was undertaken in September 2021 in the following eight databases: PubMed, EMBASE, CINAHL, Scopus, Web of Science, The Cochrane Library, PsycINFO and ProQuest. Databases were searched from inception to 11 September 2021. The database search was repeated prior to finalising the review to ensure the inclusion of articles published until 30 June 2023. No time restrictions were placed on the search and articles were not excluded based upon the date of publication alone. No language restrictions were applied during the initial literature search. The reference list of all included full text articles were hand-searched to identify any further potentially relevant studies.

Search results from the electronic databases were imported into Rayyan (rayyan.ai (accessed on 30 June 2023). Duplicates were identified and removed, followed by a final manual search of references. Studies were initially assessed by two independent reviewers (A.H. and J.O.) for inclusion by screening the title or abstract against the inclusion and exclusion criteria. Full texts of the remaining articles were then assessed for eligibility against the inclusion and exclusion criteria. Full-text articles were independently assessed for inclusion by two reviewers (A.H., J.O., L.J.S., or S.B.). Disagreement between reviewers throughout the screening process was resolved through discussion, whereby reviewers reached a consensus. The quality of these studies was assessed using the Critical Appraisal Skills Programme CASP checklist for Cohort studies [20]. Data from the included studies were recorded using a modified data collection form developed by the National Institute for Health Care Excellence (NICE). A Microsoft Excel^®^ 2021 spreadsheet was devised to collate data from eligible studies related to the author, year of the study, country, number of participants, title, duration (follow-up period), study design, inclusion and exclusion criteria, method and data collection, relevant outcomes, and key findings.

Eligibility Criteria see Table 1.

 **Quality Appraisal**  

The quality of the included studies was assessed using the Critical Appraisal Skills Programme (CASP) checklist for Cohort Studies [15]. The CASP checklist was chosen as it facilitates the assessment of the rigour, credibility and relevance of the quantitative literature [21]. Quality assessment was conducted by the author, followed by two independent reviewers. The final agreement was reached via consensus between the assessors following discussion. The results of the quality appraisal were used to moderate the findings of the review but were not used for the inclusion or exclusion of studies.

 **Data Synthesis**  

Due to the heterogeneity of the study settings, methodology and outcomes reported between studies, a meta-analysis could not be undertaken. A narrative synthesis was performed to summarise and explain the findings of the included studies [22]. Results were tabulated for summary and comparison purposes.

 **Review Criteria**  

The systematic review was conducted in accordance with the Preferred Reporting Items for Systematic Reviews and Meta-Analyses (PRISMA) Guidelines [23].

## 3. Results

### 3.1. Study Selection

**Figure 1:**
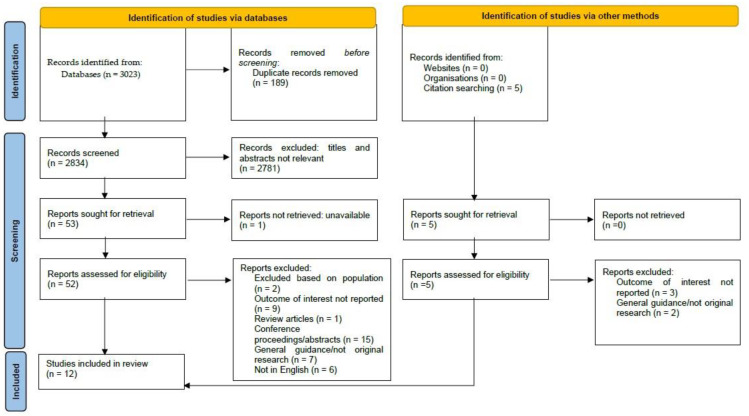
PRISMA flow diagram [23].

A total of 3023 records were identified from the initial database search. Following the removal of 189 duplicates, 2834 records remained. Following the title and abstract screen, 2781 records were excluded. A full-text review was conducted for 52 articles, with an additional 5 articles identified from hand-searching the reference lists. A review of the 57 articles against the inclusion and exclusion criteria resulted in 12 articles being included in the systematic review. The process of study selection is outlined in the PRISMA flow diagram (Figure 1).

### 3.2. Study Characteristics

#### 3.2.1. Dates of Publication

The publication dates of the included studies ranged from 2010 to 2021. Date restrictions were not applied during the search, but no earlier studies met the inclusion criteria.

#### 3.2.2. Country

The included studies were conducted in the following countries: Three studies from France [24,25,26], two studies from England [27,28], two studies from Norway [29,30], two studies from The Netherlands [31,32], and one each from Spain [33], Australia [34] and Italy [35].

#### 3.2.3. Study Design

Of the 12 studies, 7 were prospective observational studies [24,25,26,27,28,30,34], 3 were prospective observational studies with a before and after design [31,32,33], and 2 were retrospective observational studies [29,35].

#### 3.2.4. Study Participants and Settings

The number of patient participants in the 12 studies ranged from 41 [35] to 1943 [29] in the largest study. Five of the studies included patients in hospital settings (including rehabilitation and LTSUs) [24,25,26,27,35], and seven studies included patients in nursing homes [28,29,30,31,32,33,34].

#### 3.2.5. Age of Participants

Six studies provided the age of the participants, as follows; ≥65 years [24,25], >79 years [35], range 60–80 years [30], mean age 81.6 years [32], mean age 86.35 years ± 7.75 years [33]. Five studies did not provide the age of the participants, but referred to the patients as those in a geriatric unit [26], those in a stroke elderly care ward [27], those in an aged care facility [34], and psychogeriatric residents [29,31].

The characteristics of the studies included in the review are summarised in Table 2.

 **Relevant Outcomes**  

The prevalence of patients with dysphagia.

 **Results**  

The prevalence of PWDs was 16% (53/340).

Dysphagia had a negative effect on the acceptability score of oral liquids, i.e., oral liquid preparations accepted in patients without a swallowing difficulty were not accepted in those with a swallowing difficulty.
**Author; Year; Number of Participants****Study Design****Participants Included**Bourdenet et al. [25] 2015; *n* = 719 patients.Prospective, observational study.Patients ≥ 65 years, Inpatients in 23 geriatric units in Rouen University Hospital Centre (acute geriatric medicine, post -acute rehabilitation, nursing homes, long-term care units).

 **Relevant Outcomes**  

The prevalence of swallowing difficulties in hospitalised patients.

A record of the method of preparation and administration of crushed medications *.

 **Results**  

The prevalence of PWDs overall in the 23 geriatric units was 18.8% (135/719), defined as PWDs with no enteral feeding tube.

A total of 594 medications were crushed for 165 patients. An average of 3.6 medications was received by each patient who received crushed medications.

It was reported that 24.9% (148/594) were erroneously crushed.

* Note that data on the method of administration and error rate are not separate for PWDs and are reported for all patients in the study.
**Author; Year; Number of Participants****Study Design****Participants Included**Carvajal et al., 2016; [33] *n* = 1875 patients Quasi-experimental, multicentre, transversal, prospective, before-and-after observational study.Residents in 10 nursing homes.

 **Relevant Outcomes**  

The prevalence of swallowing difficulties in residents in nursing homes. The identification of patients for whom drugs were crushed. A review of the treatment to identify drugs that should not be crushed. *

 **Results**  

The prevalence of PWDs was 10% (187/1875), defined as patients with swallowing difficulties and no enteral feeding tube.

A total of 3031 medicines were administered via crushing or alteration. In total, 87.2% (2643/3031) were deemed suitable for administration following manipulation and 7.3% (220/3031) were deemed to be unsuitable for crushing.

* Note that data on the method of administration and error rate are not separate for PWDs and are reported for all patients in the study.
**Author; Year; Number of Participants****Study Design****Participants Included**Fodil et al., 2016; [26] *n* = 526 patients for prevalence survey and *n* = 143 patients for administration survey. Prospective, observational study.Inpatients with swallowing difficulties in 17 geriatric units (acute geriatric care, rehabilitation unit, long term care) of the 3 Paris-Sud teaching hospitals.

 **Relevant Outcomes**  

The prevalence of swallowing difficulties in hospitalised patients in the geriatric units studied.

The method of modification—Report upon whether national guidelines were followed.

 **Results**  

The prevalence of PWDs on geriatric inpatient units was 29.5% (155/526)—(long-term care unit 40.3% (98/243), rehabilitation unit 23.8% (46/193), acute care unit 12.2%, (11/90)).

Administration survey: (*n* = 143): Twenty patients (13.9%) had their tablets crushed as individual tablets using a mortar and pestle, and the remainder (86.1%) had their medicines crushed altogether.

It was reported that in 39 patients (27.3%), the tablets were safe to crush; however, the remainder (72.7%) of the patients had at least one medication modified where this was not performed in line with national guidelines. In the 143 patients, 110 medications were administered. In 48.2% (53/110), the modifications were not in line with national guidelines.
**Author; Year; Number of Participants****Study Design and Study Duration**
Kelly et al., 2011; [27] *n* = 625 patientsUndisguised direct observational study.Inpatients at a care-of-the-elderly ward or stroke unit at each of the four acute hospitals in the east of England.

 **Relevant Outcomes**  

The prevalence of PWDs among hospitalised patients.

The rate of Medication administration error (MAE).

 **Results**  

The prevalence of PWDs was 34.2% (214/625). There were 164 PWDs with no EFT, and 50 PWDs with an EFT in situ.

There were 2129 records of potential medicine administrations, and 31.9% (679/2129) were to PWDs. Medication administration errors (MAE) were recorded as 38.4% (817/2129), with 14.7% (313/2129) recorded for PWDs.
**Author; Year; Number of Participants****Study Design****Participants Included**Kirkevold et al., 2010; [29] *n* = 1943 residents/patients.Retrospective data collection performed by nurses. Inpatients in nursing homes in the South–East Health Region in Norway and nursing homes in the small and large municipalities in rural and urban areas.

 **Relevant Outcomes**  

Methods used to administer medicines.

Number of patients receiving an inappropriately altered medication.

 **Results**  

Of the 1943 residents, 23.3% were given at least one drug mixed into their food or beverages, and 10% (197/1943) were given at least one inappropriately altered medication.
**Author; Year; Number of Participants****Study Design****Participants Included**Mercovich et al., 2013; [34] *n* = 160 residents/patients.Prospective, observational study.Convenience sample of inpatients and consenting nurses in two aged care facilities that included high-care and dementia unit co-located facilities within the Australian Capital Territory.

 **Relevant Outcomes**  

Methods used to administer medicines and MAE rate.

 **Results**  

In total, 18% (29/160) of residents had a least one medication modified. In these 29 residents, 75 medications were modified via crushing and 32% (24/75) of these administrations were identified as not suitable for crushing according to national guidelines.

In all instances where multiple medications were administered, they were crushed together in the same vessel (using either a mortar and pestle or a pill-crushing device) and administered in the same vehicle.
**Author; Year; Number of Participants****Study Design****Participants Included**Santos et al., 2016; [28] England; *n* = 166 patients.Prospective, observational study.Inpatients with swallowing difficulties in six private care homes in North Yorkshire. Care home provided nursing care and administered medicines to patients with dysphagia or via an enteral feed tube.

 **Relevant Outcomes**  

The prevalence of PWDs among residents in nursing care homes.

The prevalence of MAE in those receiving medication.

 **Results**  

The prevalence of PWDs was 22.9% (38/166), including those with enteral feeding tubes.

Of the 738 medicine administrations that were observed in 166 patients, MAE was observed in 40.7% (300/738) of patients, of which 19.4% (143/738) was in PWDs.
**Author; Year; Number of Participants****Study Design****Participants Included**Sestili et al., 2018; [35] *n* = 41 patients at admission, *n* = 29 patients at discharge.Retrospective, medical record review. Four months. Patients hospitalised in two elderly care wards or one neurology unit in the Italian National Research Centre on Aging of Ancona. Age > 79 years Diagnosis of dysphagia carried out by the Speech and Language therapists before hospital admission or during hospitalisation.

 **Relevant Outcomes**  

The prevalence of inappropriate dosage form modifications (IDM) defined as SODFs that should not be manipulated.

 **Results**  

In total, 92% had swallowing difficulty at admission and 100% had swallowing difficulty at discharge. In total, 27% of the patients in the study had enteral feeding tubes at admission and 28% had them at discharge.

For the 41 patients, the prevalence of potentially inappropriate prescriptions PIP at admission was 41% (100/247 prescriptions), and the prevalence of IDM was 37% (37/100 prescriptions).

For the 29 patients discharged, their PIP on admission was 35% (64/185 prescriptions) and their IDM was 33% (21/64 prescriptions).
**Author; Year; Number of Participants****Study Design****Participants Included**Solberg et al., 2021; [30] *n* = 100 residents. Prospective, cross sectional, observational study.Residents in eight nursing home wards across six municipalities in mid-Norway.

 **Relevant Outcomes**  

The prevalence of SODF manipulation.

Reasons for SODF manipulation.

MAE error rate.

 **Results**  

A total of 273 administrations of a SODF were observed (each administration included 1–13 medicines). SODF modifications were performed in 20.5% (56/273) administrations, with 80.4% (45/56) involving crushing and 19.6% (11/56) involving dividing.

MAE was reported for 2.2% (6/273) of administration modifications.

The reasons for modification were swallowing difficulties in 53.6% (30/56) of instances.
**Author; Year; Number of Participants.****Study Design****Participants Included**Stuijt et al., 2012; [31] *n* = 290 for prevalence. Pre-intervention (*n* = 60) residents. First evaluation (*n* = 55) and second intervention (*n* = 62). Prospective, observational before–after design, study.Residents with swallowing difficulties in six psychogeriatric wards in two Dutch nursing homes.

 **Relevant Outcomes**  

The prevalence of patients with swallowing difficulties.

The number of MAEs per number of observed medications administered, calculated for all three subtypes of medication adverse events: crushing uncrushable medication, inappropriate technique, and food drug interactions.

 **Results**  

The prevalence of patients with swallowing difficulties: pre-intervention, 20.7% (60/290); first evaluation, 19% (55/290); and second evaluation, 21.4% (62/290).

A total of 198 observations occurred pre-intervention, 230 occurred at first evaluation, and 201 occurred at second evaluation.

MAE at pre-intervention, 23.2% (46/198) of observations; MAE at first evaluation, 15.2% (35/230) of observations; and MAE at second evaluation, 20.4% (41/201) of evaluations.
**Author; Year; Number of Participants****Study Design****Participants Included**Van Welie et al., 2016; [32] *n* = 164 patients pre-intervention and *n* = 150 patients post intervention.Prospective, uncontrolled, observational study with a pre-intervention and post-intervention measurement.Inpatients on 18 wards in 3 nursing homes in the North of the Netherlands receiving medication on an observed drug round.

 **Relevant Outcomes**  

The relative risk (RR) of a crushing error occurring in the post-intervention period compared to the pre-intervention period for PWDs. The intervention was a set of warning labels printed on each patient’s unit dose packaging indicating whether a medication could be crushed, as well as education sessions.

 **Results**  

Pre-intervention: 11.6% (19/164) of patients had their medication crushed. Error rate was 3.1% (21/681 medication administrations).

Post-intervention: 7.3% (11/150) of patients had their medication crushed. Error rate was 0.5% (3/636 medication administrations).

### 3.3. Quality Appraisal

A quality appraisal of the studies was completed and is summarised in Table 3.

### 3.4. Prevalence of Patients with Dysphagia

Seven of the twelve studies reported the prevalence of difficulties swallowing SODF, which varied from 10 to 34.2% [24,25,26,27,28,31,33]. The methods used to identify PWDs varied across the prospective observational studies. The methods used included a survey of the patients in the setting [26], identification using patient records [24,28], or physician-confirmed diagnosis [31].

PWDs sometimes had an enteral feeding tube (EFT) in place with medicines administered via the tube. Of the seven studies reporting prevalence, one reported that patients with enteral feeding tubes were excluded from the study [25], while two did not report whether these patients were excluded [26,30]. Three of the seven studies reporting prevalence included patients receiving medicines via enteral feeding tubes [27,28,33].

### 3.5. Medicines Manipulation Practices for Administration

Ten of the twelve studies reported the reason for manipulating SODFs [24,25,26,27,28,30,32,33,35]. All ten studies reported swallowing difficulties as a reason for manipulating the medicines. Nine of the twelve studies reported the methods used to manipulate SODFs [25,26,27,28,29,30,31,33,34]. The most reported method of manipulation was tablet crushing [25,26,27,29,30,33,34]. Other methods included tablet splitting [27,29,30], capsule opening [29], and mixing crushed tablet or capsule content with food [28].

### 3.6. Medication Administration Errors (MAE)

Nine of the twelve studies reported a MAE rate associated with the manipulation of SODFs to facilitate oral administration [25,26,27,28,30,34]. MAEs were identified in these nine studies by comparing the modification to recommendations contained in standard reference texts, including national guidelines and, in some instances, manufacturers’ information/Summary of Product Characteristics. Four studies calculated the MAE rate as a percentage of the number of medication administrations observed [27,28,31,32]. Three of these four studies recorded error rates for the cohort of patients and did not separate the MAE rate for PWDs. In these studies, the MAE type was confined to the erroneous crushing or altering of medicine.

One study reported that 12.7% of MAEs could have been a potential health threat and that 8.2% could have reduced efficacy because of the modification [26]. Another reported that the potential consequences of the erroneous modification of SODFs included the alteration of the drug absorption profile with the potential risk of side effects, the alteration of bioavailability and the alteration of efficacy [30]. Studies reported the inappropriate crushing of sustained-release formulations [25,26,30,32,33], the chewing of sustained-release formulations [27], the crushing of enteric-coated formulations [25,26,30,32,33], the chewing of enteric-coated formulations [28], and potential exposure to toxic drug substances following crushing [26,30,32]. The medicine groups most commonly associated with MAE were central nervous system drugs such as antipsychotics [25,26,33,34] and analgesics [25,26], cardiovascular system drugs such as dipyridamole SR [25,26,27,28,30,32,33,34], and gastrointestinal system drugs such as proton-pump inhibitors [25,27,28,30,32,33]. One study provided a comparison of MAE rates in patients with and without dysphagia [27]. PWDs were more likely than those without dysphagia to experience a MAE when analysed at a drug level: 21.1% (95% CI = 18.0–24.1) versus 5.9% (95% CI = 4.7–7.1), respectively. A higher percentage of PWDs experienced a MAE: 32.6% (95% CI = 26.2–38.9) compared to 13.8% (95% CI = 10.5–17.2) for patients without swallowing difficulties. Furthermore, PWDs with an EFT experienced higher levels of MAE compared to those without: 56% (95% CI = 42.2–69.8) versus 25.3% (95% CI = 18.6–32.0) [27].

The implications for the practice highlighted by the authors include the need for guidance, training and education for HCPs when SODFs are used for PWDs [25,26,28,30,31,32,34]. The importance of multidisciplinary working [28,30,35] and the inclusion of a pharmacist in the event that oral medicines are required for PWDs was also highlighted [27,33]. These measures assist in the mitigation of the risks of medication error. Other implications for practice included the need to assess patients’ ability to swallow medicines when a patient is admitted to a clinical area [27], understanding that organisational barriers can reduce the possibility of medicines being administered correctly [31] and that oral liquids can be a suboptimal alternative to SODFs in PWDs [24]. The ability to swallow and the palatability of an oral liquid are critical to the optimal administration of a drug to a PWD. A further challenge includes the fact that liquids may have a high sorbitol content, potentially leading to diarrhoea in the patient [36]. Finally, the liquid alternative may not be bioequivalent to the originally prescribed SODF; this is particularly important for narrow therapeutic index drugs.

## 4. Discussion

This systematic review identified and critically appraised the available evidence regarding solid oral dosage form (SODFs), e.g., tablets, and challenges regarding the oral administration of medicine to inpatients in a variety of healthcare settings, such as (1) hospitals, (2) nursing homes and (3) long-term stay units (LTSUs). The prevalence of having difficulties swallowing SODFs was found to vary from 10% to 34.2%. This means that between one to three in ten patients in hospitals, nursing homes, rehabilitation or long-term care settings have difficulty swallowing SODFs. A systematic review reported that the prevalence of having difficulties swallowing SODFs, in community-dwelling older adults aged 60 years and older, was approximately 14% [37]; it is likely that this figure is lower due to the fact that those in the community setting may not have the same level of co-morbidities associated with their care. A 2017 study reported that 3% of US hospitalised inpatients aged 45 years or older had a dysphagia diagnosis [38]. In contrast, the prevalence of swallowing difficulties was reported to be 23.9% in older patients admitted to a geriatric ward of a German community hospital [39]. The wide range of prevalence in this review may be due to the heterogeneity of the patient settings in the included studies, and the various methods used within those studies to identify PWDs. Other factors potentially influencing the prevalence range are that some studies recruited PWDs but also included other patient groups for whom medicines were modified, and that studies varied in their inclusion or exclusion of PWDs with enteral feeding tubes in situ.

Nine of the twelve studies reported the methods used to manipulate SODFs, with the most reported method being tablet crushing. Tablet splitting, capsule opening and the chewing of SODF were also reported. The access to alternative dosage forms varies according to region; for example, the UK “Specials” market allows clinicians to easily obtain unlicensed liquids for many licensed SODFs [40]. Europe, on the other hand, places greater emphasis on extemporaneous preparation and this may explain the relative necessity for manipulation between countries. Many guidelines provide advice on the suitability of SODFs for crushing or capsules for opening [36,41]. In these guidelines, there is more emphasis on SODF manipulation for administration via EFT [42] than SODF manipulation for administration to patients with swallowing difficulties without an EFT. This challenge has been recognised and a novel implicit tool, Inappropriate solid oral dosaGE form modification aSsessmenT (INGEST), has been developed [43]. This may serve to assist clinicians in determining suitability for the modification of SODFs and may help to reduce MAE. There is also a paucity of evidence to support the administration of medicines in the vehicles used to administer medicines to patients. Many SODFs are not suitable for crushing, including those with a sustained-release formulation or enteric coating. There is also a risk of exposure to the drug, which may be teratogenic or have other effects when exposed to aerosolised particles [44].

Given the concerns raised above, it is evident that MAE can occur when tablets/capsules are being administered to patients with swallowing difficulties. The most reported error was the crushing of a SODF that, due to its sustained release profile or enteric coating, should not be crushed [25,26,30,32,33]. As the actual harm from crushing SODFs is often not recognised nor reported, it would be important to include some measure of the potential harm related to MAE in future research. While there are case reports of serious negative outcomes for patients when medicines are inappropriately crushed [45], there is currently no standardised protocol to gauge these potential errors within healthcare systems.

This review has highlighted the prevalence and the methods used to modify medicines and how these modified medicines are administered. Modifying medications may affect the physical and chemical stability of the drug, as well as the clinical performance of the drug, which may lead to increased adverse effects or toxicity or decreased efficacy. These changes could potentially affect clinical outcomes for patients.

Good medication adherence is associated with improved health outcomes, with the risk of mortality amongst patients with good adherence being approximately half that of poorly adherent patients; this is particularly true in older adults [46]. The majority (69%) of patients with difficulty swallowing solid oral dosage forms in a general population reported not taking a tablet or capsule due to difficulty swallowing the dosage form [47]. Implications for practice include the need to recognise swallowing difficulty as a barrier to adherence to medication and the need for effective interventions to overcome the same. Interventions to overcome non-adherence due to swallowing difficulties could include the education of patients and healthcare professionals, the availability of guidelines on medicine administration to PWDs, and the clinical assessment of patients’ ability to swallow medicines [1]. Evidence suggests that interventions initiated at the hospital–community interface may be most effective at improving adherence [6]. Additional high-quality evidence on improving adherence in patients with dysphagia is required. Future strategies potentially able to mitigate against this may involve the proactive use of a screening tool for swallowing difficulties [1], something which may prompt healthcare providers to have a conversation with their patients and enable a more tailored approach to their oral medications.

## Figures and Tables

**Table 1:** Inclusion and exclusion criteria for the systematic review.

Inclusion Criteria	Exclusion Criteria
Studies where the results of the original research are presented.	Studies that solely involved community-dwelling patients.
Studies published in the English language. Studies investigating the oral administration of medicines to inpatients in a variety of healthcare settings, such as (1) hospitals, (2) nursing homes and (3) long-term stay units (LTSUs). Studies including patients over 18 years with swallowing difficulties. Studies in which the outcome included information on all or any of the following: (i) the prevalence of swallowing difficulties, (ii) the medication administration practices used to administer oral medicines to these patients and, where reported, (iii) the prevalence, type, classification, and severity of medication errors associated with SODF manipulation.	Studies that focused solely on medication modification for administration via an enteral feeding tube. Studies in which the medication modification was performed solely to enable covert the administration of medications. Surveys collecting data from healthcare professionals on their medicine administration practices when manipulating medicines for administration. Qualitative studies, systematic reviews, meta-analyses, conference abstracts, editorials, and commentaries.

**Table 2:** Presentation of results and key elements for each article described.

Author; Year; Number of Participants	Study Design	Participants Included
Belissa et al., 2019 [24]; *n* = 340 patients.	Multicentre, prospective, cross sectional, non-interventional observational study.	Patients ≥ 65 years, Hospitalised in any of eight French hospitals or residing in any of eight French nursing homes.

**Table 3:** Quality Appraisal using CASP—cohort study checklist [20].

	Belissa 2019 [24]	Bourdenet 2015 [25]	Carvajal 2016 [33]	Fodil 2016 [26]	Kelly 2011 [27]	Kirkevold 2010 [29]	Mercovich 2013 [34]	Santos 2016 [28]	Sestili 2018 [35]	Solberg 2021 [30]	Struijt 2012 [31]	Van Welie 2016 [32]
Did the study address a clearly focused issue?	✔	✔	✔	✔	✔	✔	✔	✔	✔	✔	✔	✔
Was the cohort recruited in an acceptable way?	✔	✔	✔	✔	✔	✔	✔	✔	✔	✔	✔	✔
Was the exposure accurately measured to minimise bias?	N/A	N/A	N/A	N/A	N/A	N/A	N/A	N/A	N/A	N/A	N/A	N/A
Was the outcome accurately measured to minimise bias?	✔	✔	✔	✔	✔	✔	✔	✔	✔	✔	✔	✔
Have the authors identified all important confounding factors?	✔	±	✔	±	✔	±	✔	±	✔	✔	✔	✔
Have they taken account of the confounding factors in the design and/or analysis?	✔	±	✔	±	✔	±	✔	±	✔	✔	✔	✔
Was the follow-up of subjects complete enough?	N/A	N/A	N/A	N/A	N/A	N/A	N/A	N/A	N/A	N/A	N/A	N/A
Was the follow-up of subjects long enough?	N/A	N/A	N/A	N/A	N/A	N/A	N/A	N/A	N/A	N/A	N/A	N/A
What are the results of this study?	✔	✔	✔	✔	✔	✔	✔	✔	✔	✔	✔	✔
How precise are the results?	N/A	N/A	N/A	N/A	✔	N/A	N/A	N/A	N/A	N/A	N/A	N/A
Do you believe the results?	✔	✔	✔	✔	✔	✔	✔	✔	✔	✔	✔	✔
Can the results be applied to the local population?	✔	✔	✔	✔	✔	✔	✔	✔	±	✔	✔	✔
Do the results of the study fit with other available evidence?	✔	✔	✔	✔	✔	✔	✔	✔	✔	✔	✔	✔
What are the implications of this study to practice?	✔	✔	✔	✔	✔	✔	✔	✔	✔	✔	✔	✔

✔ = yes, ± = can’t tell, N/A = not applicable.
